# Liver resection is associated with better survival than TACE for hepatocellular carcinoma modestly beyond Milan criteria: a multicenter propensity-matched analysis

**DOI:** 10.3389/fonc.2026.1814367

**Published:** 2026-07-24

**Authors:** Xiaoyan Fan, Guobin Sun, Yong Meng, Xiaodong Liu, Linyin Zhang, Yan Peng, Pang Wang, Liping Qiao, Jun Zhu, Rui Chen

**Affiliations:** 1 Health Examination Center, Northwest University First Hospital, Xi’an, China; 2 Department of Experiment Surgery, Xijing Hospital, Air Force Medical University, Xi’an, Shaanxi, China; 3 Department of Burns and Plastic Surgery, People’s Hospital of Ningxia, Yinchuan, China; 4 Department of General Surgery, Northwest University First Hospital, Xi’an, China; 5 Department of Stomatology, The Third Hospital of Xi’an, Xi’an, China; 6 Xijing Hospital, Air Force Medical University, Xi’an, Shaanxi, China; 7 Outpatient Department, Northwest University First Hospital, Xi’an, China; 8 Department of General Surgery, The Southern Theater Air Force Hospital, Guangzhou, China

**Keywords:** beyond the Milan criteria, hepatocellular carcinoma (HCC), liver resection (LR), liver transplantation (LT), transarterial chemoembolization (TACE)

## Abstract

**Background and aim:**

Liver transplantation (LT) is considered the optimal treatment for hepatocellular carcinoma (HCC) within extended transplant criteria, but it is available to only a minority of patients. The optimal alternative treatment strategy for patients with HCC beyond the Milan criteria (BMC) but still within extended transplant frameworks—defined in this study as modestly beyond the Milan criteria (MBMC)—remains unclear. This study aimed to compare the overall survival (OS) associated with liver resection (LR) versus transarterial chemoembolization (TACE) in patients with MBMC-HCC.

**Methods:**

Between January 2010 and October 2021, 1,110 patients with MBMC-HCC and Child–Pugh A liver function from 15 hepatobiliary centers in China were included, including 424 who underwent LR and 686 who received TACE. Overall survival was compared between groups using propensity score matching (PSM). The findings were further evaluated in additional BMC-HCC cohorts defined according to other extended transplant criteria, including the Asan criteria, up-to-7 criteria, the French AFP model, and the Metroticket 2.0 model.

**Results:**

In the multicenter MBMC-HCC cohort, as well as in the additional BMC-HCC cohorts, LR was associated with significantly longer overall survival than TACE (all log-rank P < 0.001). After 1:1 PSM, the survival advantage of LR remained consistent. Multivariable analyses and subgroup analyses across tumor burden and other baseline factors showed similar trends, although these findings should be interpreted with caution.

**Conclusions:**

LR was associated with significantly better overall survival than TACE in selected patients with MBMC-HCC. These findings support further prospective studies to clarify the role of LR in this population when transplantation is not available.

## Introduction

Hepatocellular carcinoma (HCC) is one of the most common malignancies with increasing incidence worldwide (1). Liver transplantation (LT) is currently thought as the best oncologic treatment for HCC since it replaces the diseased liver and restores normal hepatic function (2). The Milan criteria (1 lesion ≤ 5 cm or 2–3 lesions each ≤ 3 cm combined with the absence of extrahepatic lesions or macrovascular invasion) (3) have remained the benchmark in selecting candidates with HCC eligible for transplantation over the last two decades (4, 5). To increase patient access to LT, several expanded selection criteria have been advocated for HCC beyond the Milan criteria (BMC) with an appropriate overall tumor burden (6–10). Among them, the University of California, San Francisco (UCSF) downstaging protocol has recently been adopted as the standardized criteria, with initial selection criteria as 1 lesion ≤ 8 cm, 2–3 lesions each <5 cm or 4–5 lesions all < 3 cm with total tumor diameter < 8 cm (6). Substantial evidence has reported the noninferiority of these modest expansions (11, 12), although post-LT survival incrementally decreases with increasing tumor burden (13). However, regardless of whether the initial tumor meets or exceeds Milan criteria, LT is only offered to a minority of these candidates owing to limited availability of donor organs and drop-out due to tumor progression (14). Therefore, it is essential to provide effective alternative strategies for candidates who failed to receive LT.

Undoubtedly, liver resection (LR) is recommended as the preferred alternative treatment for HCCs meeting the Milan criteria (15, 16) with decent long-term survival in studies completed after 2010 and cohorts who underwent enhanced surveillance (17). However, whether LR can be performed for BMC-HCCs remains controversial (18). According to the Barcelona Clinic Liver Cancer (BCLC) classification (19, 20), single and multinodular BMC-HCCs are categorized as early-stage and intermediate-stage, respectively. For the former, LR should be preferred for single larger nodules followed by TACE if LR is infeasible (19). For the latter, transcatheter arterial chemoembolization (TACE), instead of LR, was once recommended for most intermediate HCCs according to the 2018 BCLC version (15). However, the intermediate stage is stratified into three subgroups according to tumor burden and liver function according to the latest 2022 BCLC version. Patients meeting the Extended Liver Transplant criteria (ELTC; i.e., inclusion criteria of UCSF down-staging protocol) could be candidates for LT, whereas patients without the option of transplant or matching transplant-failure but with preserved portal flow and defined tumor burden are candidates for TACE (19). Unfortunately, currently there have been few reports concerning whether LR could offer better survival for patients beyond Milan criteria but meeting ELTC.

Herein, we aimed to identify the optimal alternative option for BMC-HCC patients meeting ELTC based on a large-scale, Chinese nationwide, multicenter retrospective survey. Based on the above considerations, a particular HCC population was screen according to the following criteria: single lesion > 5 cm and ≤ 8 cm; or 2–3 lesions with at least one >3 cm and ≤ 5 cm with total tumor diameter ≤8; or 4–5 lesions with none > 3 cm with total tumor diameter ≤8, which were defined as modestly beyond Milan criteria (MBMC) in the present study. The survival benefit of LR versus TACE in the MBMC-HCC cohort was compared based on a propensity score matching analysis and was further validated in other BMC-HCC cohorts selected according to other extended criteria for LT including the Asan criteria (7), up-to-7 criteria (10), French alpha-foetoprotein (AFP) model (8) and Metroticket 2.0 model (9), respectively. We hope that our research will provide reliable guidance on treatment decisions for patients with MBMC-HCC who fail to receive a LT.

## Methods

### Study design

**Figure 1:**
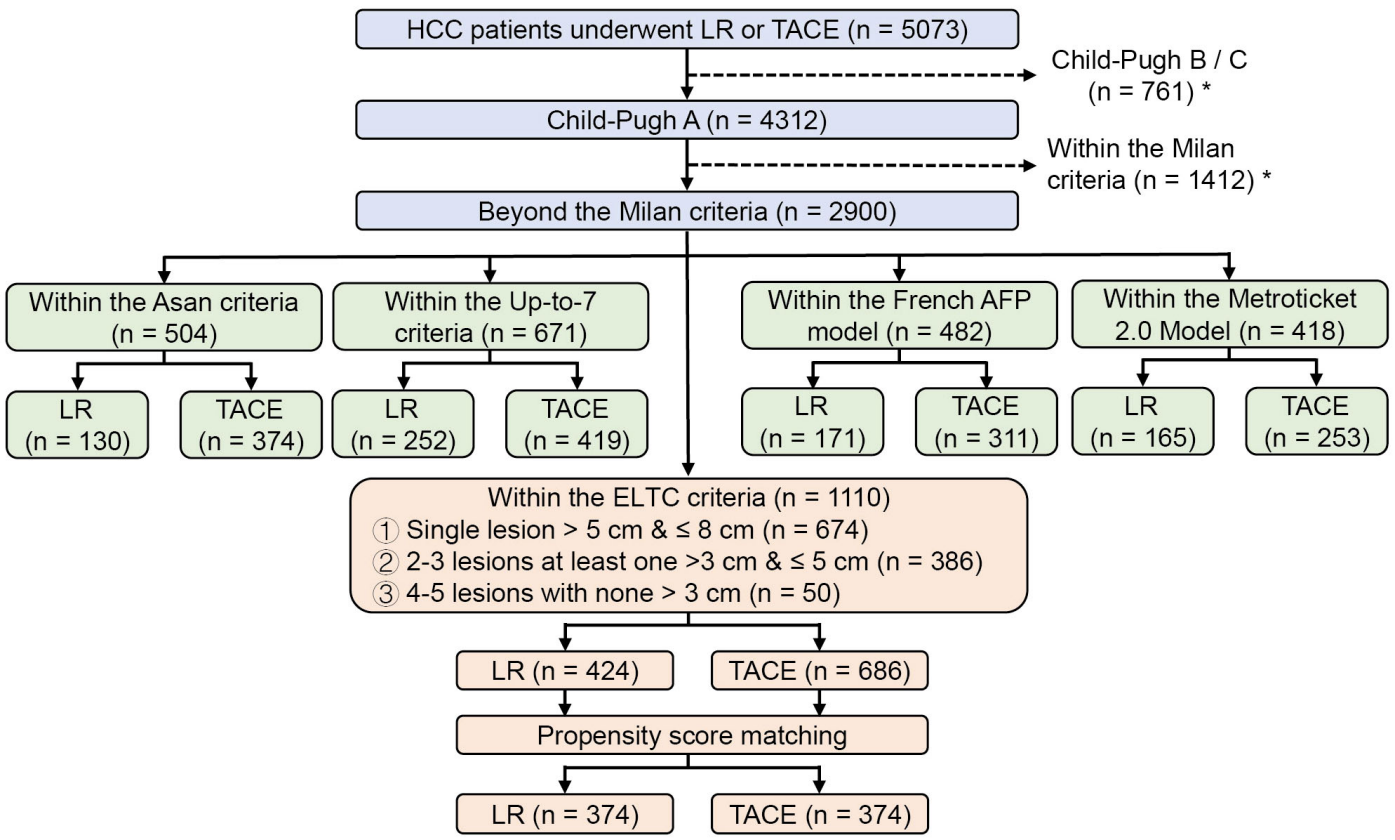
Flow chart of the study design. Asterisks represent excluded cases. HCC, hepatocellular carcinoma; LR, liver resection; TACE, transarterial chemoembolization. AFP, α-fetoprotein; ELTC, Extended Liver Transplant Criteria.

Between January 2010 and October 2021, 5,073 patients diagnosed with HCC who underwent LR or TACE from fifteen Chinese hepatobiliary centers were enrolled. Patients beyond the Milan criteria but within ELTC were included into this study: (I) single lesion > 5 cm and up to 8 cm; (II) 2–3 lesions with at least one >3 cm and up to 5 cm; (III) 4–5 lesions with none > 3 cm (6). Patients were excluded if they met any of the following criteria: (I) Child-Pugh B or C; (II) key baseline clinical or tumor-related data required for eligibility assessment and analysis were unavailable, including Child–Pugh class, tumor size, tumor number, performance status, AFP level, INR, and other essential laboratory parameters; (III) radiological evidence of extrahepatic metastasis, portal vein or other major vascular involvement (21); (IV) receiving treatment other than LR or TACE. Ultimately, 1,110 MBMC-HCC patients (LR, 424; TACE, 686) were included in this cohort study (**Figure 1**).

In addition, 4 specifical BMC-HCC cohorts meeting other extended criteria for LT were established based on the exclusion criteria described above and their respective inclusion criteria as presented in **Supplementary Table 1** for further verification. The study was approved by the Ethics Committee of the Army Medical Center of PLA on human research (No: 2022-233) and Institutional Review Board of Tang Du Hospital, Air Force Medical University (No: TDLL-202303-11), and individual consent for this retrospective analysis was waived.

### Diagnosis and definitions

HCC diagnosis was proposed preoperatively based on at least two imaging examinations (magnetic resonance imaging, sonography, contrast-enhanced dynamic computed tomography or hepatic arterial angiography) or single imaging technique together with serum AFP level > 400 ng/mL, and was confirmed by histopathological examination of resection specimens in the LR group (22). In contrast, most patients in the TACE group were diagnosed based on imaging findings together with serum markers in accordance with routine clinical practice, while only a subset of TACE patients underwent fine-needle biopsy with histopathological confirmation. The severity of cirrhosis was determined according to the Child-Pugh classification. Performance status was determined by using the ECOG-PS of 0 (asymptomatic) to 4 (restricted to bed). Vascular invasion was defined by the presence of thrombus adjacent to the tumor in the portal vein with vague boundaries. Tumor size was defined as the maximum radial diameter of the largest invasive tumor based on the results of imaging tests.

### Treatment

The criteria for LR were based on comprehensive assessment including serum total bilirubin (TBIL) levels, indocyanine green clearance, residual liver volume estimated by volumetric computed tomography, as well as absence of ascites and hepatic encephalopathy. Multiple lesions were removed by en bloc excision or multiple excisions when patients had an adequate functional liver remnant with good liver function. TACE was performed after tumor vascularity was investigated by hepatic arteriography. During TACE, the catheter tip was inserted selectively into the tumor-feeding artery under image guidance. An injection of a mixture containing anticancer agent (doxorubicin, cisplatin, mitomycin C, epirubicin and 5-fluorouracil) and lipiodol, followed by arterial embolization using 300–500 micron gelatine sponge particles was then performed. The doses of the anticancer agent lipiodol and embolic particles were determined based on both the size and extent of the lesions.

Because of the retrospective observational design, patients were not assigned to treatment according to a predefined protocol. The choice between LR and TACE was made in routine clinical practice at each participating center according to the treating team’s judgment and the patients’ clinical characteristics.

### Follow-up

Post-treatment follow-up, including ultrasonography, enhanced computed tomography or magnetic resonance imaging of the abdomen, serum biochemistry and AFP levels, was performed every three months in the first year and twice a year in subsequent years. The prognosis of these patients was confirmed at each follow-up. Patients with tumor recurrence were treated with re-resection, radiofrequency ablation, TACE, conservative treatment, or sorafenib depending on the tumor site, liver function, and the general condition (19). Follow-up treatment for those receiving TACE included repeated TACE, local ablative treatment, or conservative treatment which was determined by the general condition and liver functional status (18). The median follow-up time was computed with censored observations only. The primary outcome was overall survival (OS) measured from the date of diagnosis to death or last follow-up (for censored patients).

### Statistical analysis

Quantitative data were analyzed using the Mann-Whitney U-test, and categorical data with the chi-square test or Fisher’s exact test as appropriate. Survival curves were generated using the Kaplan-Meier method and compared using the log-rank test. The proportional-hazards assumption was evaluated using the Schoenfeld residuals test and by visually inspecting the log(-log(Survival)) curves. Univariate and multivariate analyses were performed using the Cox proportional hazards model. To address the varying hazard of early surgical mortality versus long-term oncological outcomes, a 90-day landmark analysis was conducted.

To overcome patient selection bias, a PSM analysis was performed using a logistic regression model according to a 1:1 nearest-neighbor method using nearest-neighbor matching without replacement, with a caliper width of 0.2. The following covariates were selected in this model: age, sex, hepatitis virus infection, ECOG-PS, Child–Pugh score, tumor number, tumor size, AFP, TBIL, aspartate transaminase (AST), alanine aminotransferase (ALT), platelets, albumin, hemoglobin, international normalized ratio (INR), blood urea nitrogen (BUN), and serum creatinine (Scr). The absolute standardized mean difference (ASMD) up to 0.1 indicating successful balance. Missing data were handled by multiple imputation with missing values in matching variables by chained equations (MICE) with 5 imputed datasets.

All tests were two-tailed, and *P* value < 0.05 was considered statistically significant. All statistical analyses were performed using SPSS software (version 22.0) and R software (version 3.3.1).

## Results

### Characteristics of all study patients

The patient enrolment scheme and research design were illustrated in **Figure 1**. 1110 MBMC (i.e. beyond the Milan criteria within the ELTC criteria) patients were identified. And additional cohorts that beyond the Milan criteria but meeting the Asan criteria, the up-to-7 criteria, the French AFP model or the Metroticket 2.0 model were respectively established (**Supplementary Table 1**).

Baseline demographics and clinical characteristics of MBMC-HCC patients who underwent LR (n = 424) or TACE (n = 686) were demonstrated in **Table 1**. Patients in the TACE group were significantly older, had more lesions as well as higher Child–Pugh scores, and were less likely to have hepatitis virus infection (all *P* < 0.001). Patients in the LR group had significantly higher ALT levels (*P* < 0.05), platelet counts (*P* < 0.001), albumin (*P* = 0.041) and hemoglobin (*P* < 0.001) levels, but had lower INR (*P* = 0.001) and Scr levels (*P* = 0.041). No significant difference in other covariates was seen between the two groups.

**Table 1:** Characteristics of MBMC-HCC patients before and after propensity score matching according to therapy.

Clinical variables	Before propensity matching				After propensity matching			
	LR (n=424)	TACE (n=686)	*P-value*	ASMD	LR (n=374)	TACE (n=374)	*P-value*	ASMD
Age, ≥ 70/< 70, n (%)	34 (8.0)/390 (92.0)	112 (16.3)/574 (83.7)	< 0.001	0.225	34 (9.1)/340 (90.9)	39 (10.4)/335 (89.6)	0.622	0.036
Gender, male/female, n (%)	374 (88.2)/50 (11.8)	595 (86.7)/91 (13.3)	0.517	0.043	328 (87.7)/46 (12.3)	327 (87.4)/47 (12.6)	1	0.008
Hepatitis virus infection, (+)/(-), n (%)	418 (98.6)/6 (1.4)	627 (91.4)/59 (8.6)	< 0.001	0.256	368 (98.4)/6 (1.6)	366 (97.9)/8 (2.1)	0.789	0.019
ECOG-PS, 1/0, n (%)	88 (20.8)/336 (79.2)	157 (22.9)/529 (77.1)	0.405	0.051	81 (21.7)/293 (78.3)	75 (20.1)/299 (79.9)	0.653	0.038
Child-pugh score, 6/5, n (%)	251 (59.2)/173 (40.8)	485 (70.7)/201 (29.3)	< 0.001	0.253	237 (63.4)/137 (36.6)	240 (64.2)/134 (35.8)	0.879	0.018
Tumor number, 1/2-3/4-5, n (%)	307 (72.4)/108 (25.5)/9 (2.1)	367 (53.5)/278 (40.5)/41 (6.0)	< 0.001	0.374	295 (69.3)/106 (28.3)/9 (2.4)	242 (64.7)/123 (32.9)/9 (2.4)	0.421	0.075
Tumor size, median (IQR)	5.80 (4.89-6.90)	5.20 (3.90-6.50)	< 0.001	0.307	5.70 (4.50-6.90)	5.60 (4.20-6.80)	0.339	0.075
AFP, ≥ 400/< 400 ng/mL, n (%)	144 (34.0)/280 (66.0)	245 (35.7)/441 (64.3)	0.561	0.037	127 (34.0)/247 (66.0)	138 (36.9)/236 (63.1)	0.445	0.061
TBIL, > 17.1/≤ 17.1 µmol/L, n (%)	162 (38.2)/262 (61.8)	246 (35.9)/440 (64.1)	0.442	0.049	140 (37.4)/234 (62.6)	135 (36.1)/239 (63.9)	0.762	0.028
AST, > 80/≤ 80 U/L, n (%)	38 (9.0)/386 (91.0)	52 (7.6)/634 (92.4)	0.429	0.052	31 (8.3)/343 (91.7)	33 (8.8)/341 (91.2)	0.896	0.02
ALT, > 80/≤ 80 U/L, n (%)	67 (15.8)/357 (84.2)	76 (11.1)/610 (88.9)	0.027	0.15	53 (14.2)/321 (85.8)	51 (13.6)/323 (86.4)	0.916	0.017
PLT, < 150/≥ 150×10 (9)/L, n (%)	184 (43.4)/240 (56.6)	453 (66.0)/233 (34.0)	< 0.001	0.478	182 (48.7)/192 (51.3)	200 (53.5)/174 (46.5)	0.214	0.1
Albumin, g/L, median (IQR)	41.50 (39.13-44.38)	41.00 (38.17-44.50)	0.041^†^	0.12	41.50 (39.10-44.10)	41.65 (38.60-44.80)	0.632^†^	0.042
Hemoglobin, g/L, median (IQR)	145.0 (134.0-154.0)	140.0 (126.0-149.0)	< 0.001^†^	0.312	143.00 (132.52-153.00)	142.00 (131.00-151.00)	0.361^†^	0.047
INR, median (IQR)	1.02 (0.96-1.10)	1.04 (0.99-1.11)	0.001^†^	0.218	1.03 (0.97-1.12)	1.03 (0.97-1.09)	0.872^†^	0.028
BUN, mmol/L, median (IQR)	5.56 (4.50-6.20)	5.30 (4.30-6.19)	0.103^†^	0.115	5.56 (4.50-6.20)	5.30 (4.30-6.27)	0.295^†^	0.003
Scr, μmol/L, median (IQR)	73.09 (66.00-80.00)	74.00 (64.45-84.32)	0.041^†^	0.157	73.09 (66.00-80.00)	73.09 (64.00-83.00)	0.581^†^	0.07

^†^ Mann-Whitney U test. ASMD indicates absolute standardized mean difference; LR, liver resection; TACE, transarterial chemoembolization; ECOG-PS, Eastern Cooperative Oncology Group performance status; AFP, alpha-fetoprotein; TBIL, total bilirubin; AST, aspartate transaminase; ALT, alanine aminotransferase; PLT, platelet; IQR, interquartile range; INR, international normalized ratio; BUN, blood urea nitrogen; Scr, serum creatinine.

Among patients treated with LR, the overall perioperative mortality was 2.1%, the 90-day mortality reached 6.2%, and post-hepatectomy liver failure occurred in 5.8%. Among patients treated with TACE, the most frequently observed adverse event was post-embolization syndrome (23.5%). In patients with early-stage HCC receiving TACE, treatment was usually delivered in 1–2 sessions, with 79.2% undergoing no more than two TACE procedures. Some patients achieved complete response after the first TACE, whereas others required additional sessions because of scheduled retreatment, residual viable lesions, or later recurrence.

### Survival analysis of all patients

**Figure 2:**
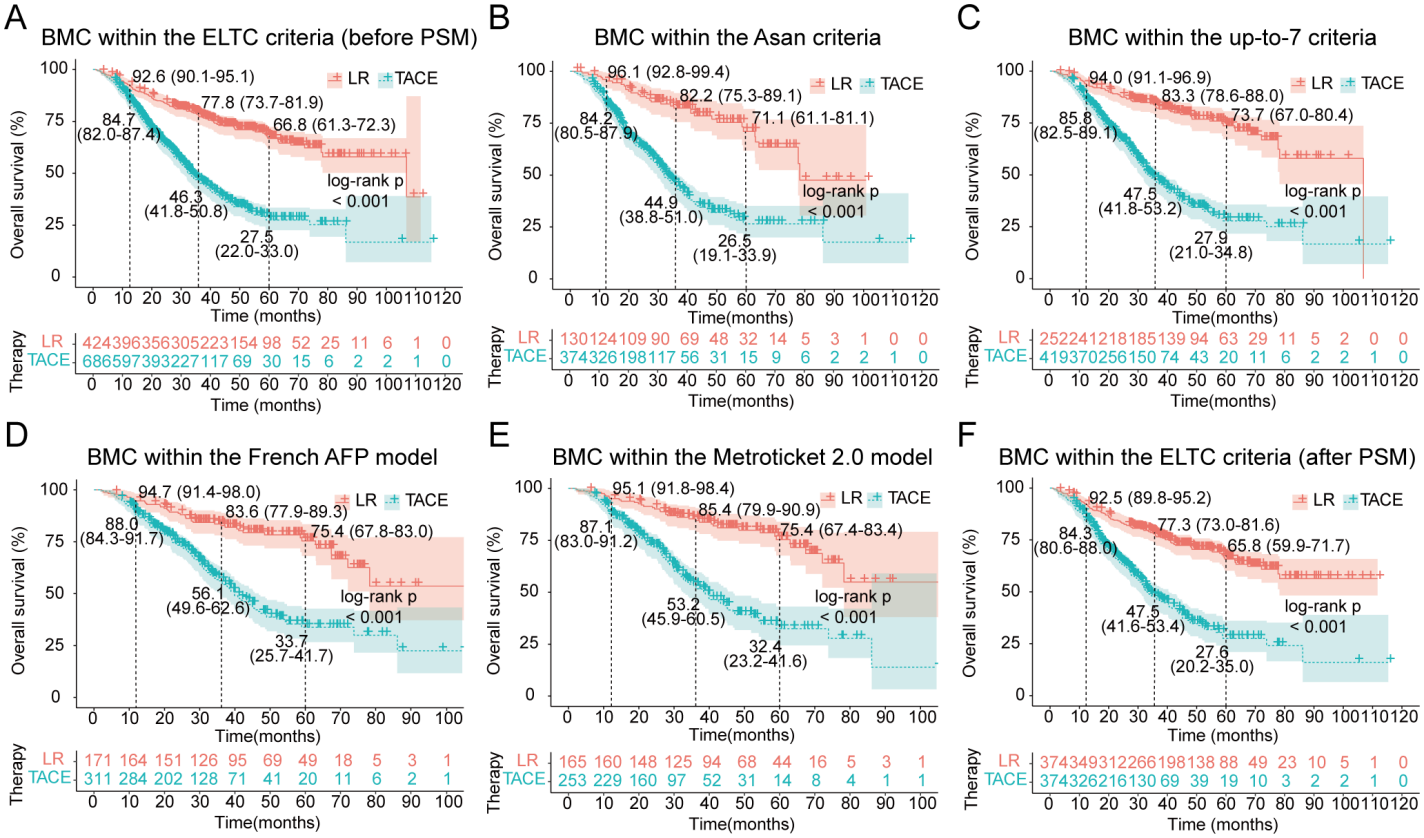
Overall survival in BMC-HCC patients within different extended criteria for liver transplantation including **(A)** the ELTC criteria, **(B)** the Asan criteria, **(C)** the up-to-7 criteria, **(D)** the French AFP model and **(E)** the Metroticket 2.0 model according to therapy (LR or TACE). **(F)** Overall survival in BMC-HCC patients within the ELTC criteria after PSM. LR, liver resection; TACE, transarterial chemoembolization; ELTC, Extended Liver Transplant Criteria; AFP, α-fetoprotein; PSM, propensity score matching.

The median follow-up time of patients in the MBMC-HCC cohort was 43.2 months (95% confidence interval [CI], 40.8-45.7), and the median OS was 51.9 months (95% CI, 44.5-59.3). The median follow-up times were 51.0 months (95% CI, 47.6-54.5) and 35.1 months (95% CI, 32.3-37.9) for the LR and TACE groups, respectively (*P* < 0.001). Furthermore, the 1-, 3-, and 5-year overall survival rates were 92.64%, 77.80%, and 66.75% in the LR group, compared to 84.66%, 46.32%, and 27.50% in the TACE group. The Schoenfeld residuals test (P = 0.12) and approximately parallel log(-log) survival curves indicated no significant global violation of the proportional-hazards assumption (**Supplementary Figure 1**). As shown in **Figure 2A**, OS in the LR group was significantly better than that in the TACE group (log-rank P < 0.001). However, given the front-loaded risk of surgical mortality, a 90-day landmark analysis was performed. For patients surviving beyond 90 days, LR was associated with significantly better OS compared to TACE (**Supplementary Figure 2**). Consistent results in the other BMC-HCC cohorts (**Figures 2B–E**; all log-rank P < 0.001) showed that LR was associated with significantly better survival than TACE in patients beyond the Milan criteria, even when different extended LT criteria were applied.

Since patients with different tumor burden were included together, we further compared the 1-, 3-, and 5-year survival rates following LR and TACE in patients with single lesion or multiple lesions (**Supplementary Figure 3**). LR and TACE for multiple lesions both offered a survival outcome comparable with that for single lesion, and LR exhibited consistently a better overall survival benefit versus TACE. Moreover, survival analyses based on the lesion number indicated that single lesion offered only a slight survival advantage over multiple lesions in BMC patients within the up-to-7 criteria (log-rank P = 0.030; **Supplementary Figure 4A**) and BMC patients within the French AFP model (log-rank P = 0.025; **Supplementary Figure 4B**). While no statistically significant difference was observed between single lesion and multiple lesions in other 2 BMC cohorts (log-rank P > 0.05; **Supplementary Figures 4C, D**). In summary, BMC-HCC patients showed similar survival outcomes across different tumor burdens within the examined cohorts. This observation may have implications for patient selection, but does not in itself demonstrate homogeneity among populations defined by different extended LT criteria.

### PSM analysis in the MBMC-HCC cohort

To reduce the potential impact of confounding factors on survival, propensity score matching (PSM) was performed to balance baseline covariates between the two groups. Among the 686 patients in the TACE group, 374 (54.5%) were successfully matched with an equal number of patients from the LR group (88.2%). After matching, baseline covariates were well balanced between the two groups, with all absolute standardized mean differences (ASMDs) ≤ 0.1. The distribution of propensity scores per group before and after matching is presented in **Supplementary Figure 5**. Among the matched patients, no statistically significant differences were observed in baseline clinical characteristics between the two groups (**Table 1**).

After PSM, the median follow-up time for the matched cohort was 45.4 months (95% CI, 42.9–48.0), and the median OS was 59.6 months (95% CI, 47.0–72.2). The median follow-up time remained significantly longer in the LR group than in the TACE group (52.2 months [95% CI, 48.8–55.7] vs. 36.0 months [95% CI, 32.2–39.9], *P* < 0.001). Notably, the matched MBMC-HCC patients who underwent LR showed significantly improved OS than those who underwent TACE (**Figures 2F**, log-rank *P* < 0.001), which is consistent with the observations in the entire MBMC-HCC cohort.

**Figure 3:**
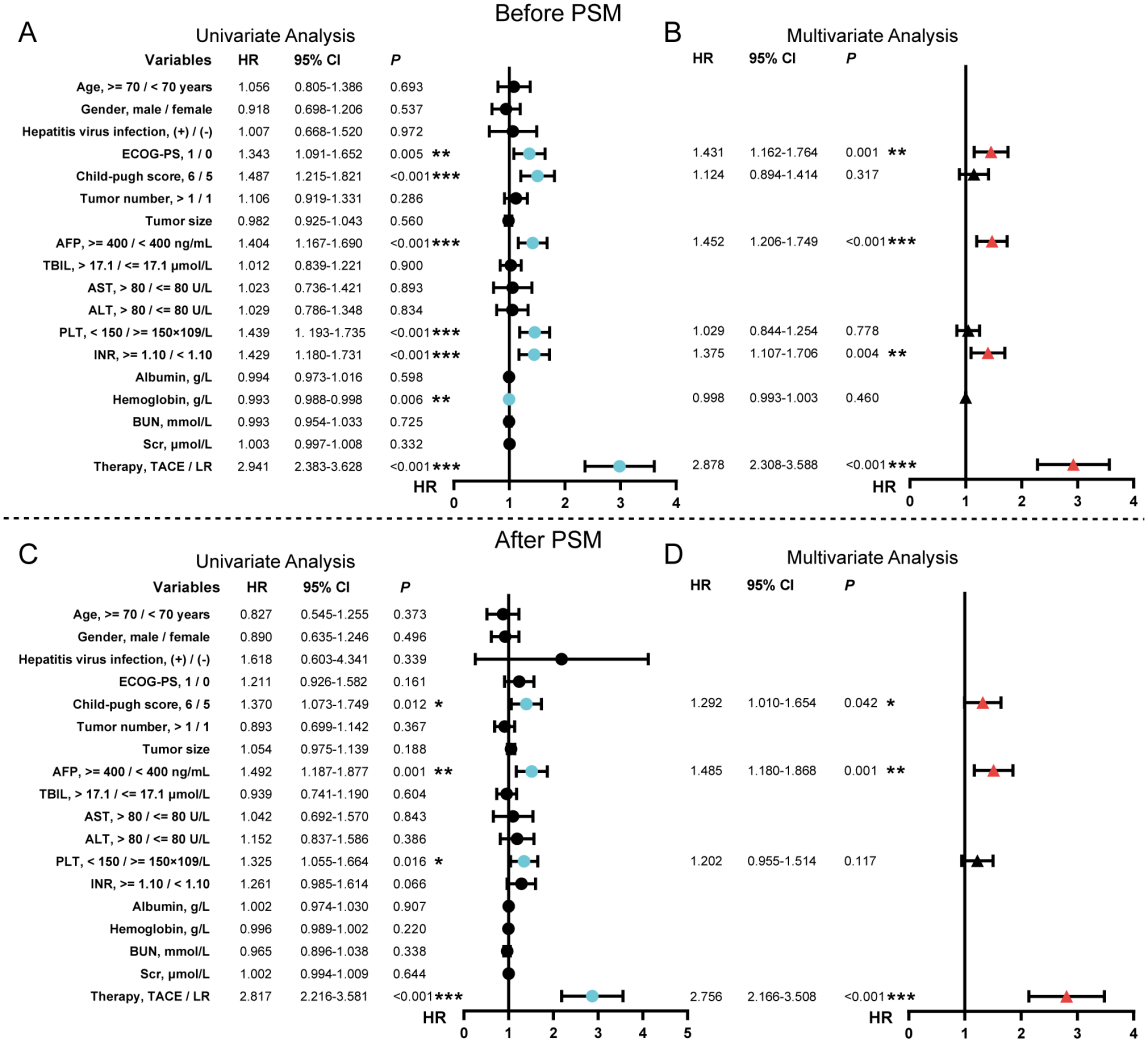
Univariate and multivariate Cox regression analyses of overall survival in MBMC-HCC patients **(A, B)** before and **(C, D)** after PSM. *P < 0.05, **P < 0.01, ***P < 0.001. PSM, propensity score matching; ECOG-PS, Eastern Cooperative Oncology Group performance status; AFP, alpha-fetoprotein; TBIL, total bilirubin; AST, aspartate transaminase; ALT, alanine aminotransferase; PLT, platelet; INR, international normalized ratio; BUN, blood urea nitrogen; Scr, serum creatinine.

To assess whether the association between treatment modality and survival was influenced by other clinical variables, univariable and multivariable Cox regression analyses were subsequently performed in the overall MBMC-HCC cohort and the propensity score-matched cohort, respectively. As shown in **Figures 3A, B**, several independent predictors of poor prognosis were identified before PSM, namely, ECOG score = 1 [hazard ratio (HR): 1.431; 95% CI, 1.162-1.764; P = 0.001), serum AFP ≥ 400 ng/mL (HR: 1.452; 95% CI, 1.206-1.749; P < 0.001), INR ≥ 1.1 (HR: 1.375; 95% CI, 1.107-1.706; P = 0.004), and TACE (HR: 2.878; 95% CI, 2.308-3.588; P < 0.001). Univariate and multivariate analyses after PSM (**Figures 3C, D**) revealed that Child-Pugh score = 6 (HR: 1.292; 95% CI, 1.010-1.654; P = 0.042), serum AFP > 400 ng/mL (HR: 1.485; 95% CI, 1.180-1.868; P = 0.001) and TACE (HR: 2.756; 95% CI, 2.166-3.508; P < 0.001) were independent predictors of poor prognosis.

### Subgroup analyses of patients matched in the propensity model

**Figure 4:**
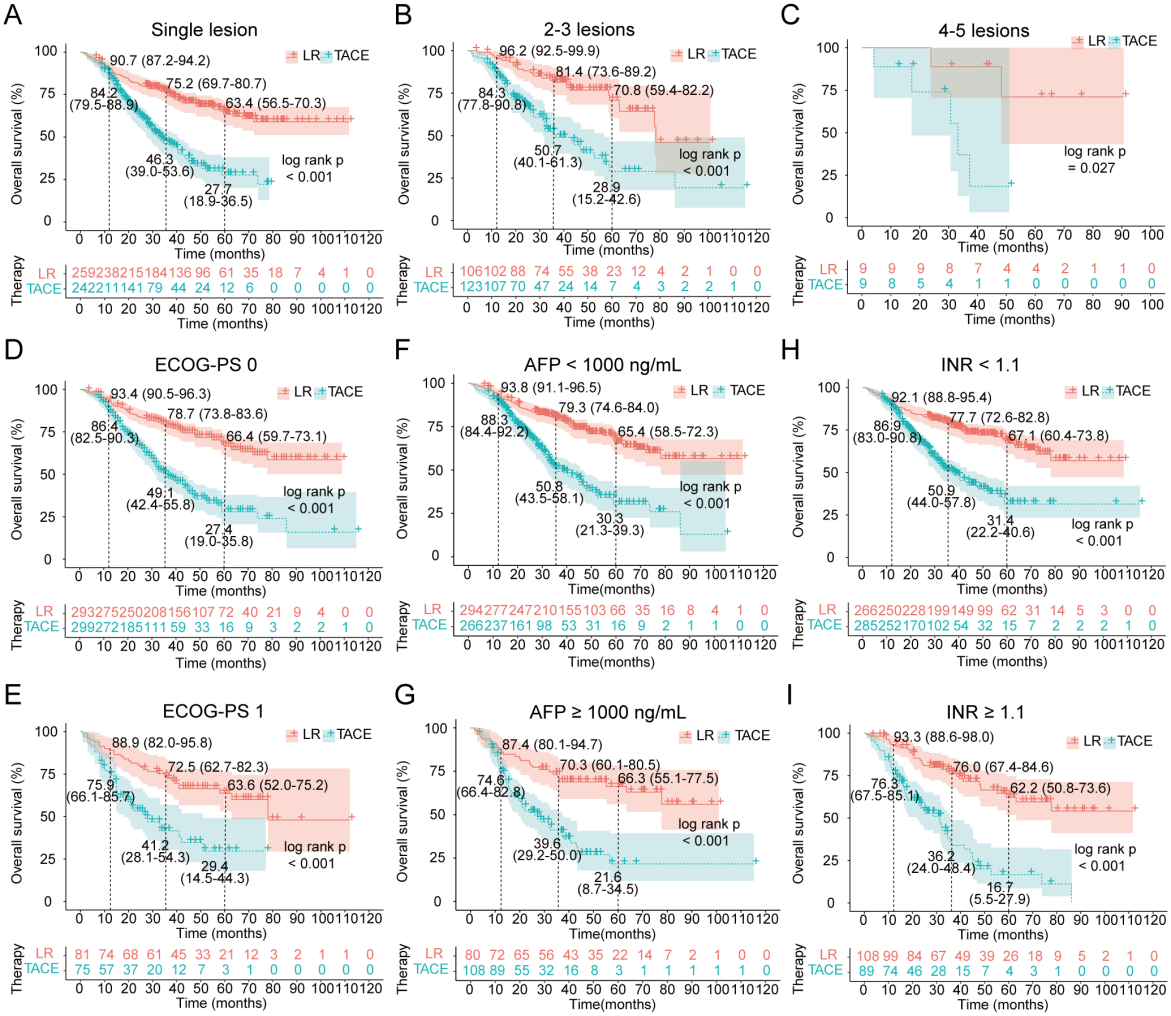
Subgroup Analysis of MBMC-HCC Patients after propensity score matching. Overall survival in patients with **(A)** single lesion, **(B)** 2–3 lesions, and **(C)** 4–5 lesions according to therapy (LR or TACE). Overall survival in patients with **(D)** ECOG-PS 0 and **(E)** ECOG-PS 1 according to therapy. Overall survival in patients with **(F)** AFP < 1000 ng/mL and **(G)** ≥ 1000 ng/mL according to therapy. Overall survival in patients with **(H)** INR < 1.1 and **(I)** INR ≥ 1.1 according to therapy. LR, liver resection; TACE, transarterial chemoembolization; ECOG-PS, Eastern Cooperative Oncology Group performance status; AFP, alpha-fetoprotein; INR, international normalized ratio.

To more deeply explore the survival benefits of LR for MBMC-HCC, subgroup analyses for OS were further performed on the basis of several characteristics. Among patients with single lesion or 2–3 lesions, the OS was significantly better in the LR group than in the TACE group (log-rank P < 0.001), and LR experienced a survival benefit over TACE at 1 year, 3 years, and 5 years (**Figures 4A, B**). Notably, even with a relatively small number of patients with 4–5 lesions, a significant survival benefit for LR over TACE was also observed (log-rank P = 0.027; **Figure 4C**). Additionally, among patients with ECOG-PS 0 or ECOG-PS 1 (**Figures 4D, E**), patients with serum AFP levels < 1000 ng/mL or ≥ 1000 ng/mL (**Figures 4F, G**), and patients with INR < 1.1 or INR ≥ 1.1 (**Figures 4H, I**), LR consistently exhibited a significantly better survival outcome compared to TACE (all log-rank P < 0.001).

Subgroup analyses based on AFP, ECOG, tumor number, and INR showed no significant interactions with treatment (all P > 0.05). Specifically, the interaction terms for AFP (P = 0.686), ECOG (P = 0.249), tumor number (P = 0.703 for 2–3 lesions, P = 0.496 for 4–5 lesions), and INR (P = 0.107) were not statistically significant. Therefore, treatment effects were not significantly modified by these baseline factors.

## Discussion

In this large-scale multicenter retrospective study, LR was associated with improved overall survival compared with TACE in patients with MBMC-HCC and preserved liver function. These findings were derived from propensity score-matched analyses and were further evaluated in additional BMC-HCC cohorts defined according to different extended LT criteria. Univariable and multivariable Cox regression analyses consistently identified treatment modality, Child–Pugh score, and serum AFP level as independent factors associated with overall survival. Subgroup analyses based on tumor burden, ECOG-PS, AFP, and INR showed similar trends across the examined subgroups, although these findings should be interpreted with caution.

According to the latest BCLC guideline, resection or ablation is the first treatment option for single HCCs if the patient has contraindications or no access to LT. TACE is only recommended if resection or ablation is unsuccessful or not feasible (23). Despite these strategies, current understandings concerning the treatment of single large HCCs are still lacking. Early data have documented that patient with HCC > 5 cm had a worse 5-year survival than those with HCC ≤ 5 cm after LR (24). However, recent studies reported positive outcomes after resection even in single large HCC (≥ 5 cm) (23) even huge HCC (≥ 10 cm), and demonstrated that LR should not be considered based on solely the tumor burden (25). However, these studies had limitations, such as small sample sizes, lack of comparison groups, and/or selection bias. Herein, we observed that LR provided a 5-year survival rate of 63.4% in single large HCCs (> 5 cm and up to 8 cm), and exhibited a significant better OS over TACE. In support of prevailing guidelines (15, 16), we advocated LR for candidates with large solitary MBMC-HCCs who failed to accept a LT.

TACE, as a non-curative option, was once recommended as the first-line treatment for intermediate-stage HCC (19, 20). However, clinicians are becoming more aware that TACE should be considered more cautiously in potentially resectable patients. It was reported that LR for intermediate or advanced HCCs were associated with high risks of intraoperative bleeding and postoperative liver failure (26). Nevertheless, with advances in surgical techniques and perioperative management, LR had been strongly advocated for HCC beyond early-stage in several institutions due to improved long-term survival (18, 27, 28). In the present study, we further investigate the OS of LR versus TACE for multinodular MBMC-HCCs in our large cohort, and demonstrated the significantly better survival benefit of LR. One interesting phenomenon in our results was that no significant difference was observed in OS among patients with different tumor burdens and tumor number or size was not an independent factor for poor prognosis. Subgroup analyses also suggested that LR provides a comparable survival advantage in single or multiple MBMC-HCCs. These data seemed to contradict with prior researches indicating the independent prognostic value of tumor number or size for HCC (29, 30). We believe that this discrepancy may be due to the narrow eligibility criteria of our study population. Meanwhile, the comparable prognosis observed in patients with single versus multiple MBMC-HCCs suggests that survival outcomes may be relatively consistent across different tumor-burden subgroups within this cohort.

Moreover, our multivariate analyses in MBMC-HCCs revealed several independent prognostic factors for OS, including ECOG-PS, Child score, AFP and INR. Thus, in addition to tumor size and number, many other factors need to be considered when making treatment decisions. Subgroup analyses based on ECOG-PS, AFP, and INR showed that LR was associated with improved survival compared with TACE in patients with MBMC-HCC across the subgroups examined.

This study has several limitations. First, due to its retrospective multicenter design, treatment selection was based on real-world clinical practice rather than a predefined protocol, and residual confounding cannot be completely excluded despite the use of propensity score matching and multivariable adjustment. Second, clinically relevant variables related to resectability and prognosis—such as indocyanine green clearance, future liver remnant, portal hypertension, tumor location/distribution, bilobar involvement, and center-level variation—were not uniformly available and could not be included in the propensity score model. Third, heterogeneity across participating centers in surgical techniques, perioperative management, follow-up strategies, and treatment indications may have influenced outcomes. In addition, because the study period spanned more than a decade (2010–2021), temporal changes in imaging, perioperative care, TACE techniques, systemic therapies, and supportive care may also have affected treatment allocation and survival outcomes; however, these era effects were not explicitly modeled. Fourth, important clinical endpoints such as recurrence, progression, liver decompensation, and treatment-related safety outcomes were not consistently available across centers. Fifth, the median follow-up was shorter in the TACE group both before and after PSM. This follow-up imbalance may have reduced the precision and statistical power of survival estimation in the TACE arm and should be considered when interpreting the results. Finally, a substantial proportion of TACE patients were excluded during propensity score matching, which may limit generalizability, particularly for the TACE cohort.

In conclusion, LR was associated with better OS than TACE across the tumor-burden subgroups examined in this study among carefully selected patients with MBMC-HCC. Given the retrospective design and the potential for residual confounding, these findings should be interpreted cautiously and warrant prospective validation.

## Data Availability

The raw data supporting the conclusions of this article will be made available by the authors, without undue reservation.
