# Supplementary material for: Liver resection is associated with better survival than TACE for hepatocellular carcinoma modestly beyond Milan criteria: a multicenter propensity-matched analysis
Source: Front Oncol. 2026 Jul 24;16:1814367. doi: 10.3389/fonc.2026.1814367 (PMC13447154; doi:10.3389/fonc.2026.1814367)
Supplement: Supplementary file 1 [file DataSheet1.docx]

**Supplementary Materials**

**Figure S1.**	The log(-log) survival curves for LR and TACE groups

**Figure S2. The** 90-day landmark analysis for LR and TACE groups.

**Figure S3.** The 1-, 3-, and 5-year survival rates following LR (red) or TACE (green) in BMC-HCC patients within different extended criteria for liver transplantation including (A) the ELTC criteria, (B) the up-to-7 criteria, (C) the French AFP model and (D) the Metroticket 2.0 model according to lesion number (single lesion or multiple lesions). Sizes of circles are proportional to the number of cases; numbers in parenthesis indicate 95% confidence intervals. Data outside brackets indicate the percentage of survival; data inside brackets indicate 95% confidence interval. YSR, year survival rate; LR, liver resection; TACE, transarterial chemoembolization; ELTC, Extended Liver Transplant Criteria; AFP, α-fetoprotein.

**Figure S4.** Overall survival in BMC-HCC patients within different extended criteria for liver transplantation including (A) the up-to-7 criteria, (B) the French AFP model, (C) the Metroticket 2.0 model and (D) the ELTC criteria according to lesion number (single lesion or multiple lesions). LR, liver resection; TACE, transarterial chemoembolization; ELTC, Extended Liver Transplant Criteria; AFP, α-fetoprotein.

**Figure S5.** The distribution of propensity scores in the LR (red) and TACE (blue) groups of MBMC-HCC patients (A) before and (B) after PSM. PSM, propensity score matching; LR, liver resection; TACE, transarterial chemoembolization.

**Table S1** Inclusion criteria of 4 BMC-HCC cohorts

| **Cohorts** | **Inclusion criteria** | **Total cases**  **N (%)** ^†^ | **Cases with single lesion**  **N (%)**^†^ | **Cases with multiple lesions**  **N (%)** ^†^ |
| --- | --- | --- | --- | --- |
| **BMC within the Asan criteria** | (1) 2-3 lesions with at least one > 3 cm and up to 5 cm; (2) 4-6 lesions with none > 5 cm. | Total: 504 (100%) LR: 130 (25.8%) TACE: 374 (74.2%) | Single lesion: 504 (100%) LR: 130 (25.8%) TACE: 374 (74.2%) | None |
| **BMC within the up-to-7 criteria** | (1) Single lesion > 5 cm and up to 6 cm; (2) 2 lesions with at least one > 3 cm and up to 5 cm; (3) 3 lesions with at least one > 3 cm and up to 4 cm; (4) 4 lesions with none > 3 cm; (5) 5 lesions with none > 2 cm; (6) 6 lesions with none > 1 cm. | Total: 671 (100%) LR: 252 (37.6%) TACE: 419 (62.4%) | Single lesion: 288 (42.9%) LR: 144 (21.5%) TACE: 144 (21.5%) | Multiple lesions: 383 (57.1%) LR: 108 (16.1%) TACE: 275 (41.0%) |
| **BMC within the French AFP model** | (1) Single lesion > 5 cm and up to 6 cm; (2) 2-3 lesions with at least one > 3 cm and up to 6 cm; (3) at least 4 lesions with none > 3 cm; AFP in all cases up to 100 ng/mL. | Total: 482 (100%) LR: 171 (35.5%) TACE: 311 (64.5%) | Single lesion: 166 (34.5%) LR: 89 (18.5%) TACE: 77 (16.0%) | Multiple lesions: 316 (65.5%) LR: 82 (17.0%) TACE: 234 (48.5%) |
| **BMC within the Metroticket 2.0 model** | (1) Single lesion > 5 cm and up to 6 cm; (2) 2 lesions with at least one > 3 cm and up to 5 cm; (3) 3 lesions with at least one > 3 cm and up to 4 cm; (4) 4 lesions with none > 3 cm; (5) 5 lesions with none > 2 cm; (6) 6 lesions with none > 1 cm; AFP in all cases up to 200 ng/mL. | Total: 418 (100%) LR: 165 (39.5%) TACE: 253 (60.5%) | Single lesion: 186 (44.5%) LR: 99 (23.7%) TACE: 87 (20.8%) | Multiple lesions: 232 (55.5%) LR: 66 (15.8%) TACE: 166 (39.7%) |
| ^†^ N, number of patients; %, percentage of patients.  AFP, alpha-foetoprotein; LR, liver resection; TACE, transarterial chemoembolization. | | | | |

**TableS2** Data-contributing centers, coordinating institutions, ethics approvals, and data-use authorization status

| **Center No.** | **Data-contributing center** | **Role** | **Ethics / authorization status** |
| --- | --- | --- | --- |
| 1 | First Affiliated Hospital of Northwest University | Coordinating center; data-contributing center | No: NUEC20230415 |
| 2 | Xijing Hospital, Air Force Medical University | Coordinating center; data-contributing center | KY20232010-C-1 |
| 3 | Tang Du Hospital, Air Force Medical University | Coordinating center; data-contributing center | TDLL-202303-11 |
| 4 | Army Medical Center of PLA on human research | Coordinating center; data-contributing center | No: 2022-233 |
| 5 | People's Hospital of Ningxia | Data-contributing center | De-identified data-use authorization |
| 6 | The Third Hospital of Xi'an | Data-contributing center | De-identified data-use authorization |
| 7 | The Southern Theater Air Force Hospital | Data-contributing center | De-identified data-use authorization |
| 8 | Daping Hospital, Third Military Medical University | Data-contributing center | De-identified data-use authorization |
| 9 | Air Force Hospital, Western Theater Command | Data-contributing center | De-identified data-use authorization |
| 10 | The Affiliated Hospital of Southwest Medical University | Data-contributing center | De-identified data-use authorization |
| 11 | Shaanxi Provincial People’s Hospital | Data-contributing center | De-identified data-use authorization |
| 12 | Huazhong University of Science and Technology | Data-contributing center | De-identified data-use authorization |
| 13 | The Seventh Affiliated Hospital of Sun Yat-sen University | Data-contributing center | De-identified data-use authorization |
| 14 | The Affiliated Hospital of Southwest Medical University | Data-contributing center | De-identified data-use authorization |
| 15 | Northern Theater Command | Data-contributing center | De-identified data-use authorization |
